# Septicemia and Aortic Valve Endocarditis due to *Erysipelothrix rhusiopathiae* in a Homeless Man

**DOI:** 10.1155/2013/923034

**Published:** 2013-04-10

**Authors:** Dean Campbell, Mark Cowan

**Affiliations:** University of Maryland School of Medicine, Baltimore, MD 21201, USA

## Abstract

We report a case of bacterial endocarditis due to *Erysipelothrix rhusiopathiae* in a homeless man with no animal exposure. His course was complicated by an allergic reaction to ampicillin, urinary bladder infection, respiratory failure, and acute kidney injury. He recovered completely after aortic valve replacement and a 6-week course of intravenous ceftriaxone.

## 1. Background

*Erysipelothrix rhusiopathiae* is a gram-positive rod causing swine erysipelas. It is a zoonotic infection in humans, with meat (swine) and fish handlers being at greatest risk. It most commonly causes erysipeloid, a localized cellulitis caused by direct bacterial invasion of cuts or abrasions in the skin. However, the skin infection can become generalized, and the organism can produce acute systemic septicemia. We report the case of a patient with *E. rhusiopathiae* bacteremia complicated by renal failure, respiratory failure, and aortic valve endocarditis.

## 2. Case Presentation

A 51-year-old Caucasian man without significant past medical history presented to a community hospital with a two-week history of shortness of breath and new onset chest pain. These symptoms were accompanied by the appearance of a rash on his fingers that spread up to his hands and wrists, but which had resolved before presentation to health care. Physical exam was significant for fever to 38.1°C, but otherwise normal vital signs. There was diffuse rhonchi heard bilaterally and a diastolic murmur heard best at the heart base. Significant laboratory values included: WBC count 14,500/mm^3^ with 40% segmental neutrophils and 44% bands, hematocrit 25.4%, blood urea nitrogen 96 mg/dL, and serum creatinine 2.4 mg/dL. The liver function tests were normal, as was the coagulation profile. Urinalysis was significant for hematuria with >100 white blood cells. Leukocyte esterase was positive. Electrocardiogram showed a normal sinus rhythm without conduction abnormalities, and his initial chest X-ray was normal. 

The patient had a history of moderate alcohol use. He had a remote history of intravenous heroin abuse, although he had not used in 20 years. He was homeless, lived in his car, and worked as a mechanic. He denied any exposures to pigs or fish, although he occasionally encountered deer and rabbits in the woods where he lived. He denied eating any undercooked meat. 

The patient was pancultured, and intravenous ampicillin-sulbactam was initiated. On the fourth hospital day, the lab reported that the blood cultures were growing a gram-positive rod they could not identify, and the cultures were sent to a reference laboratory for analysis. A transthoracic echocardiogram revealed a thickened aortic valve with a possible vegetation, and moderate aortic insufficiency. The patient's respiratory status declined over the next 2 days, and he required intubation and mechanical ventilation. Chest X-ray demonstrated evolving pulmonary edema. He was then transferred to our academic medical center for further workup and management. 

On transfer the patient remained febrile. HEENT exam was significant for poor dentition and a normal fundus. Neck exam revealed elevated jugular venous pressure while lying flat and on positive pressure ventilation. Lung sounds were coarse bilaterally. Cardiac exam revealed a III/IV diastolic murmur heard best at the left lower sternal border. There were no splinter hemorrhages, Osler's nodes, or Janeway lesions. Electrocardiogram was normal. Computed tomography of the chest revealed large bilateral pleural effusions and pulmonary edema consistent with pulmonary edema, without evidence of septic emboli. A transesophageal echocardiogram showed multiple aortic valve vegetations with severe aortic regurgitation. The reference laboratory identified the gram-positive rod as *Erysipelothrix rhusiopathiae*. The patient developed an allergic reaction to ampicillin manifested as a maculopapular rash across his chest and was switched to intravenous ceftriaxone. He underwent an uncomplicated aortic valve replacement with a bioprosthetic valve. Surgical cultures were sent and were negative. The patient was sent to a long-term care facility to finish a six-week course of intravenous antibiotics and for rehabilitation. He made a full recovery and was in good health on followup visit 8 months later.

## 3. Discussion

*Rhusiopathiae* (formerly *insidiosa*) is the sole pathogenic member of the genus *Erysipelothrix*, which also includes the species *tonsillarum* and a third as yet unnamed species [1, 2]. It was first isolated by Koch in 1880 [3] and was described as the causative agent in swine erysipelas in 1886 [4, 5]. It was recognized as a pathogenic microorganism in humans in 1909 when Rosenbach described its isolation from the cutaneous lesions of erysipeloid [6]. It is the causative agent in a number of agriculturally important diseases in pigs, turkeys, chickens, ducks, shellfish, emus, and sheep. In humans, several distinct clinical syndromes have been described, and the organism is generally considered an occupational disease resulting from contact with infected animals or their waste products [2].

### 3.1. Epidemiology

 The most important reservoir of *E. rhusiopathiae* in human infection is thought to be swine, although birds and rodents are frequently infected, and many different types of animal may carry the organism, including insects. It has a worldwide distribution with isolates detected in culture from Africa, Asia, Australia, the Americas, and Europe. The organism is shed by diseased swine in all bodily fluids, even if the animal is clinically well, with an average of 20–40% of healthy swine harboring the organism, usually detected in the tonsils (oropharynx) or the feces [2]. In the environment, the organism can remain viable for up to two weeks in water, several months in picked bacon or smoked ham [7], and long periods of time in exterior fish slime [8], contaminated soil, or animal carcasses [9]. The most important risk factor is occupational exposure to animals likely to harbor the organism, as seen in farmers, butchers, veterinarians, fishermen, slaughterhouse workers, abattoir workers, and housewives [1, 7]. Other less common affected occupations include meat inspectors, knackers, animal caretakers, lobstermen, bone button makers, game handlers, fertilizer workers, cooks, seal and whale hunters, crabbers, bakers, furriers, leather makers, soap makers, and stockyard workers [8]. Seafood workers appear to be especially at risk [9]. Infection is usually through scratches or puncture wounds in the skin, although penetration through intact skin has been reported [10]. Additionally, infection by *E. rhsiopathiae* may be underdiagnosed due to the resemblance it bears to other infections, as well as the difficulty in isolating or identifying the pathogen [11]. Human-to-human transmission has not been documented.

### 3.2. Bacteriology

Morphologically, *E. rhusiopathiae* is a thin, pleomorphic, nonsporulating gram-positive rod [3]. It is nonmotile, cannot ferment sucrose, and forms clear colonies [2]. It is mildy *α*-hemolytic, and a facultative anaerobe [12]. It requires various amino acid additives, as well as riboflavin and small amounts of oleic acid to grow [13]. The organism is negative for catalase, oxidase, methyl red, indole, and Voges-Proskauer reactions [14]. More recent detection methods have used an API Coryne system strip [15] or PCR-based techniques [16]. These studies are generally performed only at a reference laboratory and on specific request by the referring hospital or physician. Immune evasion by *E. rhusiopathiae* can take two distinct forms. In the absence of specific host antibodies (i.e., de novo infection), the organism is able to evade phagocytosis by immune cells. This may be due to formation of a heat labile capsule by the organism, which has been implicated as a virulence factor in mice [17]. In the presence of specific antibodies, the organism can continue to replicate intracellularly, despite having undergone phagocytosis by immune cells [18]. It has been shown that neuraminidase plays a significant role in bacterial attachment and subsequent invasion into host cells [11]. The mechanism for this is not known. Sensitivity testing of strains of *E. rhusiopathiae* from nine pigs and one human were performed by Venditti et al. [19], and Fidalgo et al. [20]. They demonstrated good susceptibility of the organism to penicillin, imipenem, cefotaxime, ceftriaxone, piperacillin, clindamycin, and fluoroquinolones. 6/10 isolates were highly resistant to vancomycin; 4/10 were intermediate. Teicoplanin and daptomycin were somewhat better than vancomycin but were judged by the authors to be unsatisfactory. 60–80% were inhibited by erythromycin, tetracycline, and chloramphenicol. There was no activity with trimethoprim-sulfamethoxazole or aminoglycoside antibiotics. These studies imply that *β*-lactam antibiotics are the drugs of choice for the organism, with fluoroquinolones as an acceptable alternative in lactamase allergic or intolerant patients. The resistance of the organism to vancomycin occurs via intrinsic resistance rather than acquired resistance and relies on the *vanC* gene [21]. This has potentially important clinical consequences. The Gram stain, appearance, and catalase negativity may initially suggest *Lactobacillus*, *Actinomyces*, *Corynebacterium* (Diphtheroids), *Streptococcus*, or even *Enterococcus* species. Not all of these species are fully characterized in all labs, and therefore *E. rhusiopathiae* may be missed. Since the Gram stain shows a gram-positive rod, clinicians may be led to choose vancomycin empirically, and unless the organism is identified, they may inadequately treat the infection [22].

### 3.3. Clinical Characteristics

*E. rhusiopathiae* infection in humans takes three common forms [1, 2]. Most commonly, a mild, cutaneous infection termed erysipeloid forms in the area of the inoculation. It is seen after an incubation period of ~4 days (range 1–7 d). Most cases occur in the summer and early fall and affect men more often than women. Reported ages range from 10 to 72 years old (mean 45). The lesion lasts from 2 to 4 weeks and is self-limiting. A more severe cutaneous form can occur, associated with a diffuse, purpuric rash, which is blue or purple and has well-defined, raised borders. There is pain and pruritis, and the rash has a predominantly peripheral distribution. The most severe form of the disease occurs typically with a subacute onset. Preceding rash is often reported, and pharyngitis may be associated with the prodrome if consumption of infected material was the mechanism of transmission [23]. Blood cultures are generally positive for the organism, and characteristically there is concomitant endocarditis [7, 24], although this is not universal [25, 26]. 60% of the cases involve the aortic valve. Valve replacement is necessary in 35%, and mortality is 40% despite early recognition and appropriate antibiotics [27]. There can be perivalvular and myocardial abscesses [28, 29]. Physicians must have a high index of suspicion for *Erysipelothrix* to avoid empirically prescribing ineffective agents such as vancomycin and aminoglycosides [30]. It has been associated with acute leukemia in a child [31], and septicemia in a neonate [32]. There are accounts of occurrence in adults with lupus [33, 34], HIV [35], oropharyngeal cancer [36], necrotizing fasciitis [37], colon perforation [38], and several reports of association with septic arthritis [39–41]. Demonstrated consequences of septicemia include acute renal failure [42] and multiple brain infarctions [43]. Recently, there has also been a reported case of *Erysipelothrix* pneumonia in an immunocompetent patient, who likely contracted the illness from feeding his cow in a barn while smoking. The authors of the study suggested that although there have not yet been reports of *Erysipelothrix* entering the body via inhalation, the pneumonia likely developed via inhalational transmission of the organism [44].

## 4. Conclusion

*Erysipelothrix rhusiopathiae* is an uncommon cause of septicemia and endocarditis. Awareness of this organism is imperative, as proper microbiologic testing is essential in the diagnosis, and appropriate antibiotic choices can only be made through identification of the organism. It most commonly causes a self-limited skin infection, but as seen in our case, can cause life-threatening illness. 
